# Ammonium ytterbium(III) diphosphate(V)

**DOI:** 10.1107/S1600536808039664

**Published:** 2008-11-29

**Authors:** Karla Fejfarová, Rachid Essehli, Brahim El Bali, Michal Dušek

**Affiliations:** a Institute of Physics, Na Slovance 2, 182 21 Praha 8, Czech Republic; b Department of Chemistry, Faculty of Sciences, University Mohammed 1st, PO Box 717, 60 000 Oujda, Morocco

## Abstract

The title compound, NH_4_YbP_2_O_7_, crystallizes in the KAlP_2_O_7_ structure type and consists of distorted YbO_6_ octa­hedra and bent P_2_O_7_^4−^ diphosphate units forming together a three-dimensional network. There are channels in the structure running along the *c* axis, where the NH_4_^+^ cations are located. They are connected *via* N—H⋯O hydrogen bonds to the terminal O atoms of the diphosphate anions.

## Related literature

Isotypic compounds were reported by Man-Rong *et al.* (2005 ▶), [NH_4_LuP_2_O_7_]; Horchani-Naifer & Férid (2007 ▶), [YbP_2_O_7_]; Jansen *et al.* (1991 ▶), [CsYbP_2_O_7_], that all crystallize with the KAlP_2_O_7_ structure type (Ng & Calvo, 1973 ▶). For the crystal structures of other isoformular rare earth diphosphates, see: Hamady & Jouini (1996 ▶), [NaYP_2_O_7_]; Férid *et al.* (2004 ▶), [NaEuP_2_O_7_]; Ferid *et al.* (2004 ▶ ▶), [NaYbP_2_O_7_]; Férid & Horchani-Naifer (2004 ▶), [NaLaP_2_O_7_]; Horchani-Naifer & Férid (2005 ▶), [NaCeP_2_O_7_]; Hamady *et al.* (1994 ▶) and Yuan *et al.* (2007 ▶), [KYP_2_O_7_]. Possible applications of rare earth phosphates were discussed by Yamada *et al.* (1974 ▶); Hong (1975 ▶); Bimberg *et al.* (1975 ▶). For background on crystallographic software, see: Becker & Coppens (1974 ▶).

## Experimental

### 

#### Crystal data

NH_4_YbP_2_O_7_
*M* *_r_* = 365
Monoclinic, 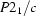

*a* = 7.6468 (2) Å
*b* = 10.9119 (2) Å
*c* = 8.6129 (3) Å
β = 105.645 (3)°
*V* = 692.04 (3) Å^3^
*Z* = 4
Mo *K*α radiation
μ = 13.97 mm^−1^
*T* = 120 K
0.26 × 0.08 × 0.07 mm

#### Data collection

Oxford Diffraction XCalibur 2  diffractometer with Sapphire 2 area detector
Absorption correction: analytical [implemented in *CrysAlis RED* (Oxford Diffraction, 2008 ▶), according to Clark & Reid (1995 ▶)] *T*_min_ = 0.169, *T*_max_ = 0.545
8574 measured reflections
1437 independent reflections
1362 reflections with *I* > 3σ(*I*)
*R*_int_ = 0.023

#### Refinement

*R*[*F* ^2^ > 2σ(*F* ^2^)] = 0.016
*wR*(*F* ^2^) = 0.061
*S* = 1.32
1437 reflections
113 parameters
4 restraints
Only H-atom coordinates refined
Δρ_max_ = 0.58 e Å^−3^
Δρ_min_ = −0.50 e Å^−3^

### 

Data collection: *CrysAlis CCD* (Oxford Diffraction, 2005 ▶); cell refinement: *CrysAlis RED* (Oxford Diffraction, 2008 ▶); data reduction: *CrysAlis RED*; program(s) used to solve structure: *SIR2002* (Burla *et al.*, 2003 ▶); program(s) used to refine structure: *JANA2006* (Petříček *et al.*, 2007 ▶); molecular graphics: *DIAMOND* (Brandenburg & Putz, 2005 ▶); software used to prepare material for publication: *JANA2006*.

## Supplementary Material

Crystal structure: contains datablocks global, I. DOI: 10.1107/S1600536808039664/wm2208sup1.cif

Structure factors: contains datablocks I. DOI: 10.1107/S1600536808039664/wm2208Isup2.hkl

Additional supplementary materials:  crystallographic information; 3D view; checkCIF report

## Figures and Tables

**Table:** 

Yb1—O1	2.240 (3)
Yb1—O2^i^	2.158 (4)
Yb1—O4^ii^	2.230 (3)
Yb1—O5	2.224 (3)
Yb1—O6^iii^	2.191 (4)
Yb1—O7^iv^	2.195 (3)
P1—O1	1.529 (3)
P1—O2	1.498 (5)
P1—O3	1.611 (4)
P1—O4	1.525 (3)
P2—O3	1.622 (4)
P2—O5	1.532 (3)
P2—O6	1.507 (4)
P2—O7	1.514 (3)

**Table:** 

P1—O3—P2	127.40 (19)

Symmetry codes: (i) 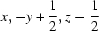; (ii) 
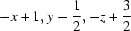
; (iii) 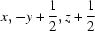; (iv) 
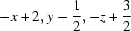
.

**Table 2:** Hydrogen-bond geometry (Å, °)

*D*—H⋯*A*	*D*—H	H⋯*A*	*D*⋯*A*	*D*—H⋯*A*
N1—H1⋯O7^v^	0.87 (4)	2.52 (5)	3.381 (5)	168 (4)
N1—H2⋯O4^vi^	0.88 (4)	2.03 (5)	2.888 (5)	166 (4)
N1—H3⋯O5^vii^	0.87 (4)	2.00 (4)	2.873 (6)	177 (7)
N1—H4⋯O1	0.86 (5)	2.26 (4)	2.916 (5)	132 (4)

Symmetry codes: (v) 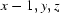; (vi) 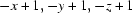; (vii) 
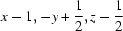
.

**Fig. 1.**
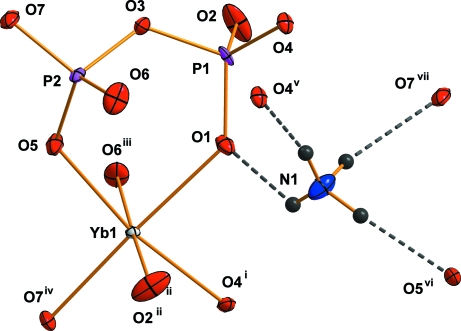
Part of the structure of NH4YbP2O7. Displacement ellipsoids are drawn at the 50% probability level. [Symmetry codes: (i) 1 - x,-1/2 + y, 1.5 - z; (ii) x, 0.5 - y, -1/2 + z; (iii) x, 0.5 - y, 1/2 + z; (iv) 2 - x, -1/2 + y, 1.5 - z; (v) 1 - x, 1 - y, 1 - z; (vi} -1 + x, 0.5 - y, -1/2 + z; (vii) -1 + x, y, z.]

**Fig. 2.**
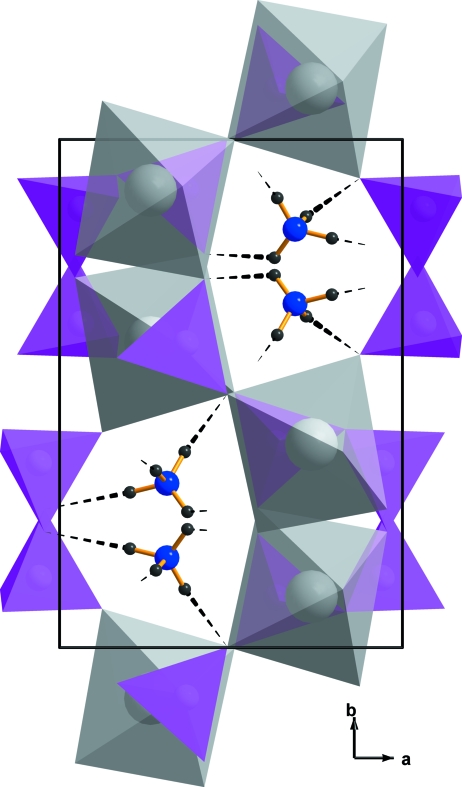
The packing of NH4YbP2O7 viewed along c. Colors: Pink (P2O7), grey (YbO6), blue balls (N), black balls (H).

## supplementary crystallographic information

### Comment

Rare earth phosphates have many potential applications in the field of optical materials including laser phosphors (Yamada *et al.*, 1974; Hong, 1975; Bimberg *et al.*, 1975). Their crystal structures depend on the ionic radii of the alkali metal and the rare earth ions. The two *A*YbP_2_O_7_ (*A* = Cs (Jansen *et al.*, 1991), K (Horchani-Naifer & Férid, 2007)) structures known so far belong to the KAlP_2_O_7_ structure type (Ng & Calvo, 1973) and crystallize in space group *P*2_1_/*c*. For the correspondent isoformular sodium rare earth diphosphates, several other structures have been described, for instance NaYP_2_O_7_ in space group *P*2_1_ (Hamady & Jouini, 1996), NaLnP_2_O_7_ (Ln = Eu (Férid, Horchani & Amami, 2004), Yb (Ferid *et al.*, 2004)) in space group *P*2_1_/*n*, and NaLnP_2_O_7_ (Ln = La (Férid & Horchani-Naifer, 2004), Ce (Horchani-Naifer & Férid, 2005) in space group *Pnma*. KYP_2_O_7_ is dimorphic and can adopt the KAlP_2_O_7_ structure type (Yuan *et al.*, 2007), or a structure in space group *Cmcm* (Hamady *et al.*, 1994).

In the present paper we report the crystal structure of NH_4_YbP_2_O_7_. This compound is isotypic with NH_4_LuP_2_O_7_ (Man-Rong *et al.* 2005), KYbP_2_O_7_ (Horchani-Naifer & Férid, 2007) and CsYbP_2_O_7_ (Jansen *et al.*, 1991). The Yb atom is coordinated by six oxygen atoms forming a distorted octahedron that belong to five symmetry-related P_2_O_7_^4-^ anions (Fig. 1). The average Yb—O bond lenght is 2.206 Å (Table 1). The diphosphate anion is bent with a bridging angle of 127.40 (19) °. The three-dimensional network of YbO_6_ and P_2_O_7_^4-^ units forms channels running along the *c* direcion in which the NH_4_^+^ cations are located (Fig. 2). Each NH_4_^+^ cation is connected *via* N—H···O hydrogen bonds to four different P_2_O_7_^4-^ anions (Table 2).

### Experimental

Three solutions have been mixed in a beaker to prepare the title compound: NH_4_OH (20 ml, 0.1 mmol), YbCl_3_^.^6H_2_O (20 ml, 0.1 mmol) and  Na_4_P_2_O_7_ (20 ml, 0.1 mmol). The pH of the mixture was controlled with diluted hydrochloric acid to be slightly acidic, and the solution was stirred for two hours at room temperature. Crystals suitable for X-ray analysis were formed after a few days.

### Refinement

All hydrogen atoms were discernible in difference Fourier maps and could be refined to reasonable geometry. The N—H distances were restrained to 0.87 Å with σ of 0.02. The isotropic atomic displacement parameters of all hydrogen atoms were refined with 1.2×*U*_eq_ of the N atom.

### Crystal data

**Table:**

NH_4_YbP_2_O_7_	*F*_000_ = 668
*M**_r_* = 365	*D*_x_ = 3.502 Mg m^−^^3^
Monoclinic, *P*2_1_/*c*	Mo *K*α radiation λ = 0.71073 Å
Hall symbol: -P 2ybc	Cell parameters from 8929 reflections
*a* = 7.6468 (2) Å	θ = 2.8–26.5º
*b* = 10.9119 (2) Å	µ = 13.97 mm^−^^1^
*c* = 8.6129 (3) Å	*T* = 120 K
β = 105.645 (3)º	Prism, colorless
*V* = 692.04 (3) Å^3^	0.26 × 0.08 × 0.07 mm
*Z* = 4	

### Data collection

**Table:**

Oxford Diffraction XCalibur 2 with Sapphire 2 area detector diffractometer	1437 independent reflections
Radiation source: X-ray tube	1362 reflections with *I* > 3σ(*I*)
Monochromator: graphite	*R*_int_ = 0.023
Detector resolution: 8.3438 pixels mm^-1^	θ_max_ = 26.5º
*T* = 120 K	θ_min_ = 2.8º
Rotation method data acquisition using ω scans	*h* = −9→9
Absorption correction: analytical[implemented in CrysAlis RED (Oxford Diffraction, 2008), according to Clark & Reid (1995)]	*k* = −13→13
*T*_min_ = 0.169, *T*_max_ = 0.545	*l* = −10→10
8574 measured reflections	

### Refinement

**Table:**

Refinement on *F*^2^	Only H-atom coordinates refined
*R*[*F*^2^ > 2σ(*F*^2^)] = 0.016	Weighting scheme based on measured s.u.'s *w* = 1/[σ^2^(*I*) + 0.0016*I*^2^]
*wR*(*F*^2^) = 0.061	(Δ/σ)_max_ = 0.039
*S* = 1.32	Δρ_max_ = 0.58 e Å^−^^3^
1437 reflections	Δρ_min_ = −0.50 e Å^−^^3^
113 parameters	Extinction correction: B-C type 1 Lorentzian isotropic (Becker & Coppens, 1974)
4 restraints	Extinction coefficient: 170 (60)
4 constraints	

### Special details

**Table:**

Refinement. The refinement was carried out against all reflections. The conventional *R*-factor is always based on *F*. The goodness of fit as well as the weighted *R*-factor are based on *F* and *F*^2^ for refinement carried out on *F* and *F*^2^, respectively. The threshold expression is used only for calculating *R*-factors *etc*. and it is not relevant to the choice of reflections for refinement.The program used for refinement, Jana2006, uses the weighting scheme based on the experimental expectations, see _refine_ls_weighting_details, that does not force *S* to be one. Therefore the values of *S* are usually larger than the ones from the *SHELX* program.

### Fractional atomic coordinates and isotropic or equivalent isotropic displacement parameters (Å^2^)

**Table:**

	*x*	*y*	*z*	*U*_iso_*/*U*_eq_	
Yb1	0.73470 (3)	0.100256 (15)	0.753623 (18)	0.00555 (9)	
P1	0.63175 (18)	0.40147 (9)	0.81812 (14)	0.0080 (4)	
P2	0.93914 (15)	0.36312 (10)	0.68708 (13)	0.0070 (3)	
O1	0.5777 (4)	0.2746 (3)	0.7457 (4)	0.0124 (10)	
O2	0.6416 (6)	0.4080 (3)	0.9940 (5)	0.0254 (14)	
O3	0.8335 (4)	0.4300 (3)	0.8037 (4)	0.0126 (9)	
O4	0.5107 (4)	0.5010 (3)	0.7202 (3)	0.0097 (9)	
O5	0.9555 (4)	0.2277 (3)	0.7359 (4)	0.0121 (10)	
O6	0.8260 (6)	0.3855 (3)	0.5169 (5)	0.0183 (12)	
O7	1.1241 (4)	0.4235 (3)	0.7273 (4)	0.0142 (10)	
N1	0.3131 (6)	0.3233 (4)	0.4381 (5)	0.0183 (13)	
H1	0.281 (7)	0.348 (5)	0.523 (4)	0.0219*	
H2	0.365 (7)	0.385 (3)	0.403 (6)	0.0219*	
H3	0.206 (4)	0.306 (5)	0.375 (5)	0.0219*	
H4	0.373 (7)	0.267 (4)	0.501 (5)	0.0219*	

### Atomic displacement parameters (Å^2^)

**Table:**

	*U*^11^	*U*^22^	*U*^33^	*U*^12^	*U*^13^	*U*^23^
Yb1	0.00611 (15)	0.00443 (15)	0.00616 (16)	−0.00018 (5)	0.00173 (9)	0.00025 (5)
P1	0.0101 (6)	0.0088 (6)	0.0056 (5)	0.0056 (4)	0.0027 (5)	−0.0006 (4)
P2	0.0066 (5)	0.0058 (5)	0.0084 (5)	−0.0016 (4)	0.0017 (4)	0.0016 (4)
O1	0.0142 (16)	0.0076 (15)	0.0178 (16)	0.0019 (12)	0.0085 (13)	0.0023 (12)
O2	0.034 (3)	0.039 (2)	0.0043 (17)	0.0210 (17)	0.0062 (17)	0.0013 (14)
O3	0.0073 (15)	0.0117 (14)	0.0158 (16)	0.0000 (12)	−0.0019 (13)	−0.0036 (13)
O4	0.0093 (14)	0.0097 (13)	0.0102 (14)	0.0026 (12)	0.0028 (11)	0.0041 (12)
O5	0.0088 (15)	0.0113 (15)	0.0166 (16)	−0.0007 (12)	0.0041 (12)	0.0045 (12)
O6	0.017 (2)	0.0277 (19)	0.0086 (18)	−0.0005 (14)	0.0015 (15)	0.0090 (14)
O7	0.0093 (16)	0.0101 (13)	0.0228 (17)	−0.0036 (13)	0.0035 (14)	0.0014 (12)
N1	0.017 (2)	0.021 (2)	0.013 (2)	−0.0069 (17)	−0.0014 (17)	0.0046 (16)

### Geometric parameters (Å, °)

**Table:**

Yb1—O1	2.240 (3)	P1—O4	1.525 (3)
Yb1—O2^i^	2.158 (4)	P2—O3	1.622 (4)
Yb1—O4^ii^	2.230 (3)	P2—O5	1.532 (3)
Yb1—O5	2.224 (3)	P2—O6	1.507 (4)
Yb1—O6^iii^	2.191 (4)	P2—O7	1.514 (3)
Yb1—O7^iv^	2.195 (3)	N1—H1	0.87 (5)
P1—O1	1.529 (3)	N1—H2	0.87 (5)
P1—O2	1.498 (5)	N1—H3	0.87 (3)
P1—O3	1.611 (4)	N1—H4	0.86 (4)
			
O1—Yb1—O2^i^	88.83 (13)	O2—P1—O3	106.3 (2)
O1—Yb1—O4^ii^	87.53 (11)	O2—P1—O4	112.5 (2)
O1—Yb1—O5	82.94 (12)	O3—P1—O4	105.70 (17)
O1—Yb1—O6^iii^	89.43 (12)	O3—P2—O5	106.33 (19)
O1—Yb1—O7^iv^	175.63 (13)	O3—P2—O6	106.2 (2)
O2^i^—Yb1—O4^ii^	91.87 (14)	O3—P2—O7	104.64 (18)
O2^i^—Yb1—O5	89.99 (15)	O5—P2—O6	113.98 (18)
O2^i^—Yb1—O6^iii^	178.23 (14)	O5—P2—O7	110.79 (17)
O2^i^—Yb1—O7^iv^	93.37 (13)	O6—P2—O7	114.1 (2)
O4^ii^—Yb1—O5	170.25 (11)	P1—O3—P2	127.40 (19)
O4^ii^—Yb1—O6^iii^	88.39 (13)	P1—O4—H2^v^	115.2 (13)
O4^ii^—Yb1—O7^iv^	88.63 (12)	P2—O5—H3^vi^	109.5 (14)
O5—Yb1—O6^iii^	89.47 (13)	H1—N1—H2	108 (5)
O5—Yb1—O7^iv^	100.82 (12)	H1—N1—H3	99 (4)
O6^iii^—Yb1—O7^iv^	88.39 (12)	H1—N1—H4	85 (5)
O1—P1—O2	113.0 (2)	H2—N1—H3	113 (4)
O1—P1—O3	107.61 (19)	H2—N1—H4	123 (5)
O1—P1—O4	111.26 (16)	H3—N1—H4	119 (4)

Symmetry codes: (i) *x*, −*y*+1/2, *z*−1/2; (ii) −*x*+1, *y*−1/2, −*z*+3/2; (iii) *x*, −*y*+1/2, *z*+1/2; (iv) −*x*+2, *y*−1/2, −*z*+3/2; (v) −*x*+1, −*y*+1, −*z*+1; (vi) *x*+1, −*y*+1/2, *z*+1/2.

### Hydrogen-bond geometry (Å, °)

**Table:**

*D*—H···*A*	*D*—H	H···*A*	*D*···*A*	*D*—H···*A*
N1—H1···O7^vii^	0.87 (4)	2.52 (5)	3.381 (5)	168 (4)
N1—H2···O4^v^	0.88 (4)	2.03 (5)	2.888 (5)	166 (4)
N1—H3···O5^viii^	0.87 (4)	2.00 (4)	2.873 (6)	177 (7)
N1—H4···O1	0.86 (5)	2.26 (4)	2.916 (5)	132 (4)

Symmetry codes: (vii) *x*−1, *y*, *z*; (v) −*x*+1, −*y*+1, −*z*+1; (viii) *x*−1, −*y*+1/2, *z*−1/2.
